# Prognostic Role of Non-Identification of Sentinel Lymph Node in Cutaneous Melanoma Patients: An Observational Retrospective Study

**DOI:** 10.3390/cancers12113151

**Published:** 2020-10-27

**Authors:** Ruggero Moro, Cintia Arjona-Aguilera, Celia Requena, Virginia Pont-Sanjuan, Victor Traves, Esperanza Manrique-Silva, Eduardo Nagore

**Affiliations:** 1 Doctorate School, Universidad Católica de Valencia San Vicente Mártir, 46001 València, Spain; ruggero.moro@mail.ucv.es (R.M.); emanrique@mail.ucv.es (E.M.-S.); 2 Dermatology, Clinica San Carlo, 20037 Paderno Dugnano, Italy; 3 Department of Dermatology, Instituto Valenciano de Oncología, 46001 València, Spain; carjona@fivo.es (C.A.-A.); crequena@fivo.es (C.R.); 4 Department of Dermatology, Hospital Arnau de Vilanova, 46001 València, Spain; vp.pont@comv.es; 5 Department of Pathology, Instituto Valenciano de Oncología, 46001 València, Spain; vtraves@fivo.es; 6 School of Medicine, Universidad Católica de Valencia San Vicente Mártir, 46001 València, Spain

**Keywords:** melanoma, lymph node biopsy, sentinel, identification

## Abstract

**Simple Summary:**

Sentinel lymph node status is the most important prognostic factor for patients with cutaneous melanoma, but occasionally it is not possible to identify the sentinel lymph node. Little is known in cutaneous melanoma literature about the phenomenon of non-identification of sentinel lymph node and its prognostic implications. In this study we observed that not identifying the sentinel lymph node involves a worse nodal disease-free survival, but not a worse melanoma-specific survival than a negative sentinel lymph node. Potentially, patients with non-identified SLN should receive a follow-up schedule like that of patients with positive SLN.

**Abstract:**

*Background*: Sentinel lymph node (SLN) status is recognized as the most important prognostic factor for patients with cutaneous melanoma. However, sometimes it is not possible to identify SLN. The phenomenon of non-identification of SLN and its prognostic role have not been thoroughly evaluated in melanoma literature. The objective of this study was to identify which patient or tumor variables may be associated to non-identification of SLN and to evaluate the prognostic role of non-identification of SLN. *Methods*: Observational retrospective study of 834 cutaneous melanoma patients who underwent SLN biopsy at Instituto Valenciano de Oncología. *Results*: Forty-two patients (5%) presented non-identification of SLN. Patients with age at diagnosis of ≥ 64 years, obesity (BMI ≥ 30), and head and neck localization were at higher risk of non-identification of SLN. Non-identified SLN patients had worse nodal disease-free survival with respect to negative SLN patients, but not worse melanoma-specific survival. *Conclusions*: Our findings suggest a need to follow-up patients with non-identified SLN in the same way as patients with positive SLN.

## 1. Introduction

Sentinel lymph node (SLN) involvement is the most important prognostic factor in patients with cutaneous melanoma [1]. Moreover, SLN status is nowadays a criterion to choose which patients with clinical stage I-II melanoma would benefit from adjuvant treatments and, also, to be eligible for clinical trials [2]. However, sometimes it is not possible to identify SLN. The phenomenon of non-identification (NI) of SLN occurs in 1–6% of SLN biopsies (SLNB), with a higher rate reported if the tumor drains to cervical or ectopic basins [3,4,5,6,7].

NI can occasionally be due to non-visualization (NV) of SLN during the preoperative lymphoscintigraphy, as NV leaves the surgeon doubting whether to attempt the SLNB, and where to attempt it in the case of an area with possible multiple lymphatic basin drainage. NV of SLN is reported in 2–3% of all melanoma patients undergoing SLNB [8,9]. Many motivations, both biological and technical, can justify NV. Lymph nodes massively replaced by metastasis, or a lymphatic drainage altered by older age or by previous interventions may impair migration of the radiotracer [10]. Moreover, incorrect radiocolloid administration, background noise, low resolution, or relying only on planar imaging may lead to failure in localizing the SLN, especially if sited on the head and neck [8].

In the same way, a difficult anatomical localization of SLN (cervical, ectopic) may cause NI, even after a successful lymphoscintigraphy. As stated above, an altered lymph flow may block the migration of the radiotracer, but also of the blue dye. The intraoperative visualization of blue-stained tissue can be critical, particularly in case of vague radiological description of SLN localization, decay of radioactive substance, or discordance between gamma camera and hand-held probe sensitivities. Finally, the surgeon’s inability to adequately identify and remove all SLNs, or an inadequate histopathological analysis may lead to NI [6,7].

The prognostic role of NI has not been thoroughly evaluated in the literature. It has been demonstrated that the prognosis of patients with thick melanoma is similarly poor in patients with positive SLN and in patients in whom SLNB is not performed [11,12,13].

In our study, we more exhaustively analyzed the phenomenon of NI of SLN in cutaneous melanoma and its prognostic role, comparing non-identified SLN patients with those with positive or negative SLN. The objectives of the study were: (1) identify which patient or tumor variables may be associated to non-identification of SLN and (2) evaluate the prognostic role of non-identification of SLN.

## 2. Results

### 2.1. Patient Characteristics

Of 2196 patients with primary cutaneous melanoma registered in the database, 834 underwent SLNB and, thus, were eligible for further analysis. Forty-two (5%) of them presented NI of SLN. Median age and Breslow thickness in the study population were 55 years (interquartile range [IR] = 42–67) and 1.65 mm (IR = 1.0–3.0), respectively.

The main characteristics of the study population and their correlation with SLN status are shown in Table 1.

### 2.2. SLN Identification

Logistic regression showed age at diagnosis (≥64 vs. <64 years; odds ratio [OR] = 3.0; confidence interval [CI] 95% = 1.6–5.7; *p* = 0.001), BMI (≥30 vs. <30; OR = 2.7; CI 95% = 1.3–5.8; *p* = 0.009), anatomical localization (head and neck vs other localization; OR = 12.2; CI 95% = 5.8–25.6; *p* < 0.001), and histological type (lentigo maligna melanoma [LMM] vs non-LMM; OR = 7.5; CI 95% = 3.2–18.0; *p* < 0.001) as predictors of NI of SLN (Table 2).

In the multivariate analysis, only anatomical localization (head/neck vs other location; OR = 17.5; CI 95% = 7.1–43.1; *p* < 0.001), BMI (≥30 vs. <30; OR = 3.8; CI 95% = 1.6–9.0; *p* = 0.002), and age at diagnosis (≥64 vs. <64; OR = 2.9; CI 95% = 1.3–6.6; *p* = 0.009) were statistically associated with NI of SLN. In this model, 240 (29%) cases were excluded from the analysis due to missing information about BMI. Thus, a second multivariate analysis was performed excluding BMI, where age at diagnosis (≥64 vs. <64; OR = 2.2; CI 95% = 1.1–4.2; *p* = 0.021) and anatomical localization (head/neck vs other location; OR = 12.2; CI 95% = 5.8–25.6; *p* < 0.001) were once again independent predictors of NI of SLN (Table 2).

### 2.3. Disease Free Survival (DFS)

After a median follow-up of 73 months (IR = 34–117), 186 patients presented disease recurrence (22.3%): 75 out of 174 positive SLN (43.1%), 100 out of 618 negative SLN (16.2%), and 11 out of 42 non-identified SLN (26.2%). Of these 186 patients, 29 showed nodal recurrence (3.5%): 14 positive SLN (8%), 11 negative SLN (1.8%) and 4 non-identified SLN (9.2%); while 86 presented distant recurrence (10.2%): 35 positive SLN (20.1%), 45 negative SLN (7.3%), and 6 non-identified SLN (14.3%).

**Figure 1:**
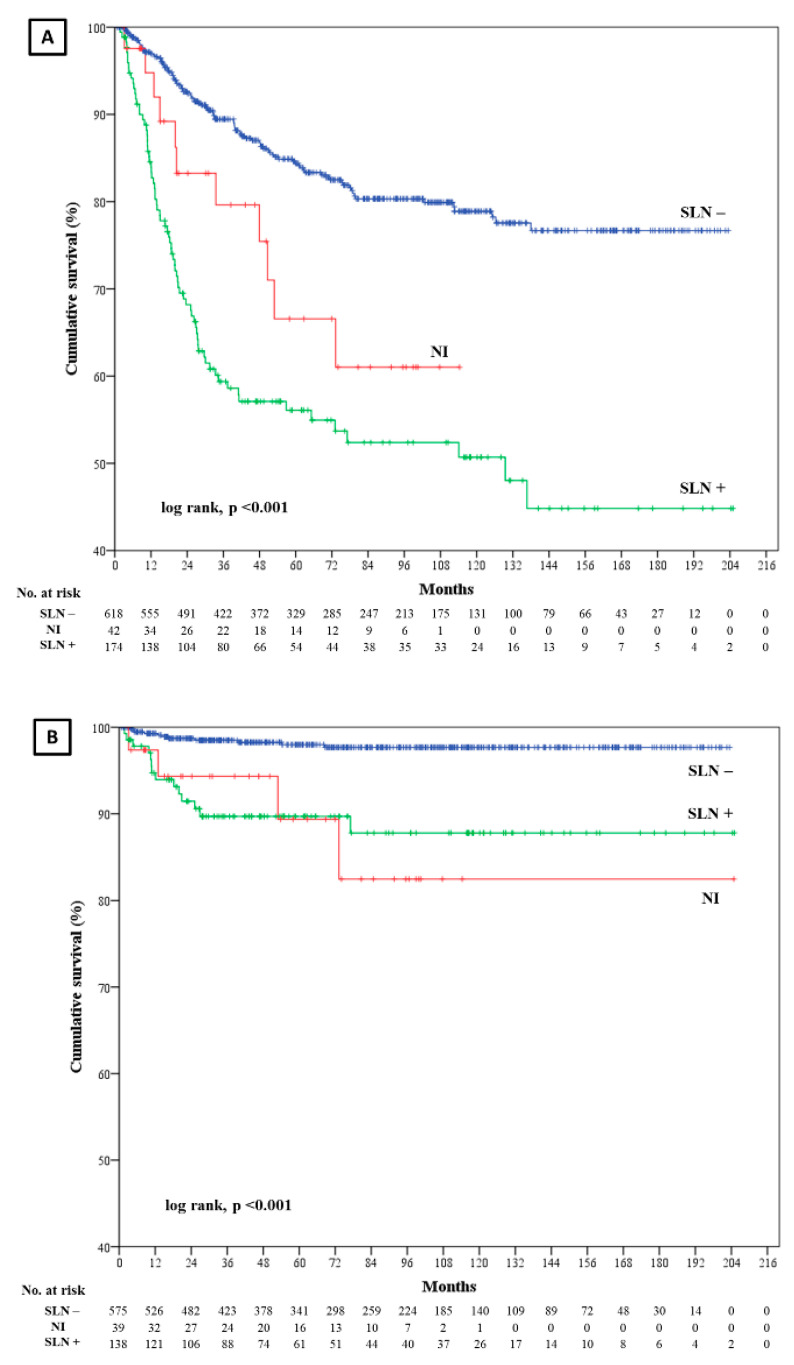
Kaplan-Meier curves depicting disease-free survival (**A**) and nodal disease-free survival (**B**) per sentinel lymph node (SLN) status. NI = non-identified.

DFS was significantly shorter in patients with positive SLN and non-identified SLN than in patients with negative SLN (log rank, *p* < 0.001) (Figure 1). The univariate model of Cox regression confirmed this result: positive SLN vs negative SLN (hazard ratio [HR] = 3.5; CI 95% = 2.6–4.7; *p* < 0.001); non-identified SLN vs negative SLN (HR = 2.1; CI 95% = 1.1–4.0; *p* = 0.018). The stepwise forward multivariate Cox proportional hazards analysis showed a worse DFS in patients with positive SLN (HR = 2.9; CI 95% = 1.7–4.7; *p* < 0.001), but not in patient with non-identified SLN (HR = 1.7; CI 95% = 0.8–4.1; *p* = 0.269) as compared to those with negative SLN. Furthermore, DFS was shorter if Breslow ≥2 mm (HR = 5.5; CI 95% = 2.2–13.6; *p* < 0.001), microscopic satellites were present (HR = 4.8; CI 95% = 1.8–13.1; *p* = 0.002) and the melanoma was localized on the head and neck (HR = 2.2; CI 95% = 1.2–4.0; *p* = 0.007).

Nodal DFS was worse in non-identified and positive SLN patients than negative SLN patients (log rank, *p* < 0.001) (Figure 1). Univariate Cox regression: non-identified SLN vs negative SLN (HR = 6.3; CI 95% = 2.0–19.7; *p* = 0.002); positive SLN vs negative SLN (HR = 5.8; CI 95% = 2.6–12.7; *p* < 0.001). Multivariate Cox regression: non-identified SLN vs negative SLN (HR = 5.1; CI 95% = 1.6–16.2; *p* = 0.006) and positive SLN vs negative SLN (HR = 3.2; CI 95% = 1.4–7.5; *p* = 0.006). Also, nodal DFS was shorter if Breslow ≥ 2 mm (HR = 4.2; CI 95% = 1.7–10.4; *p* = 0.002) and vascular invasion was present (HR = 3.7; CI 95% = 1.2–11.2; *p* = 0.022) (Table 3).

### 2.4. Melanoma Specific Survival (MSS)

A total of 116 patients died of melanoma (13.9%), of whom 54 were positive SLN (31%), 57 were negative SLN (8.2%), and five were non-identified SLN (11.9%).

**Figure 2:**
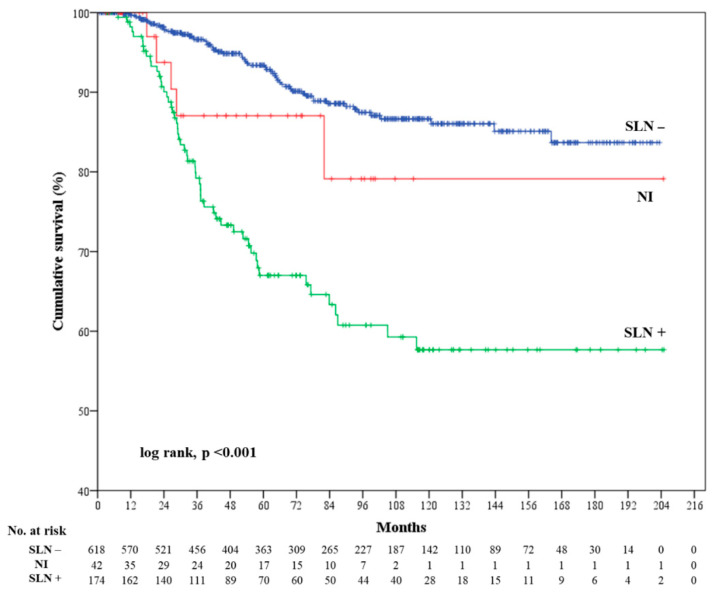
Kaplan–Meier curve representing melanoma-specific survival per sentinel lymph node (SLN) status. NI = non-identified.

MSS was significantly shorter in positive SLN patients than in negative SLN patients, though not in non-identified SLN patients (log rank, *p* < 0.001) (Figure 2). Univariate Cox regression: positive SLN vs. negative SLN (HR = 4.1; CI 95% = 2.8–5.9; *p* < 0.001); non-identified SLN vs negative SLN (HR = 1.8; CI 95% = 0.7–4.5; *p* = 0.21). Multivariate Cox regression further confirmed the above results: positive SLN vs negative SLN (HR = 2.9; CI 95% = 1.9–4.3; *p* < 0.001), non-identified SLN vs negative SLN (HR = 1.2; CI 95% = 0.5–3.1; *p* = 0.665). Moreover, patients with Breslow ≥ 2 mm (HR = 3.2; CI 95% = 2.0–4.7; *p* < 0.001), age at diagnosis ≥ 64 years (HR = 2.3; CI 95% = 1.6–3.3; *p* < 0.001), and microscopic satellites (HR = 2.7; CI 95% = 1.3–5.7; *p* = 0.009) showed a worse MSS (Table 3).

## 3. Discussion

In this study, we analyzed 834 patients affected by primary cutaneous melanoma who underwent SLNB at our institution. Forty-two (5%) of these patients presented NI of SLN. This rate is in accordance with those reported in previous studies [3,4,5,6,7]. Age at diagnosis of ≥64 years, obesity (BMI ≥30) and head and neck localization were significantly associated with NI of SLN (Table 2). Indeed, an identification rate decrease was already observed depending on the lymphatic drainage basin explored; the highest rates were reported for the inguinal site and the lowest for the cervical area [4]. Moreover, NI of SLN is statistically less frequent in recent studies, in studies with a greater proportion of ulcerated tumors, in studies with better quality scores, and in female patients [5]. If only the head and neck localization is considered, the identification rate of the SLN improves in more recent and larger studies, and in studies with a greater mean Breslow depth and a greater proportion of ulcerated tumors [3]. These data suggest that the experience and practice of the operators increase identification rates, but also that more aggressive tumors enable easier identification of SLN. It can be hypothesized that these melanomas may be associated with greater inflammation at the site of the primary tumor, leading to draining lymph node reactivity and, finally, to the development of reactive enlarged nodes that may be more easily identifiable than the smaller nodes normally found in the cervical drainage area [3].

Occasionally, NI may be caused by NV of SLN during the preoperative lymphoscintigraphy, because NV leaves the surgeon with doubt regarding whether to attempt the SLNB, and where to attempt it in case of an area with possible multiple lymphatic basin drainages. Two recent studies published NV rates of 2.3% and 3%, and consequent NI rates of 1.49% and 1.11%, respectively [8,9]. NV was facilitated by older age of the patient, head and neck localization of the melanoma, and previous operation in adjacent fields [8,9]. Obesity was not reported to be associated with NV, though higher BMI is known to impair visualization of SLN in breast cancer patients [14,15,16]. Thus, it is logical to assume that obesity may lead to NV of SLN in melanoma patients. As a matter of fact, our data showed how obesity (BMI ≥30) could impair the identification of SLN.

Other biological reasons may lead to failure of the preoperative lymphoscintigraphy: if the lymph flow is blocked by metastatic cells or if the lymphatic drainage has been altered by previous interventions or by older age, visualization of SLN may be impaired [10]. On the other hand, these biological anomalies may also lead to NI, because they may block the migration of blue dye and impair the intraoperative identification of blue-stained tissue.

Moreover, various technical factors (e.g., incorrect radiocolloid administration, background noise, low resolution, relying only on planar imaging) can compromise the visualization of SLN during lymphoscintigraphy, especially if the tumor is located next to the draining lymph node, as frequently occurs in melanomas of the head and neck [8]. Equally, many technical motivations could justify NI of SLN: a vague description of SLN localization, the decay of radioactive substance, a difficult anatomical localization of SLN (cervical, ectopic), the discordance between gamma camera and hand-held probe sensitivities, the surgeon’s inability to adequately identify and remove all SLNs intraoperatively, an inadequate histopathological analysis, and, finally, the surgeon’s decision to not attempt SLNB after NV during preoperative lymphoscintigraphy [6,7].

All these factors explain why SLNB success requires an experienced and dedicated multidisciplinary team of physicians. In the near future, the use of SPECT/TC and MRI during lymph node mapping might improve the detection rate of SLN, especially if located on the head and neck [17,18,19]. This could allow to uncover metastatic lymph nodes undetectable with planar images only, and, consequently, achieve a better DFS [18].

In our study, 186 patients presented disease recurrence, 29 in the locoregional nodal basins and 86 as distant metastases, while 116 patients died of melanoma.

DFS was shorter for positive or non-identified SLN patients as compared to those with negative SLN. Multivariate Cox regression corroborated this result for positive SLN patients (HR = 2.9, *p* < 0.001), but not for non-identified SLN patients (HR = 1.7; *p* = 0.269).

Nodal DFS was also worse in patients with non-identified or positive SLN than in patients with negative SLN (Figure 1). Remarkably, non-identified SLN patients had the highest HR (5.2; *p* = 0.006) to develop nodal recurrence, higher than patients with positive SLN (HR = 3.2; *p* = 0.006) or with Breslow ≥ 2 mm (HR = 4.2; *p* = 0.002) (Table 3). Such a result may be explained by the very same motivations that may lead to SLNB failure. For example, metastatic cells may block the lymph flow and, thus, compromise SLN identification; however, these cells may migrate and produce metastasis on the lymph node. Another reason may be that all the patients with positive SLN were treated with complete lymph node dissection (CLND), while non-identified SLN patients underwent only closer ultrasonographic follow-up. Effectively, although CLND does not improve overall survival and MSS of melanoma patients, it still permits a slightly better regional disease control [20,21,22]. Our data seemed to confirm this occurrence. Probably, some of the patients in which SLNB failed to identify the SLN would have benefit, in terms of nodal DFS, from CLND, because they were harboring occult nodal metastases.

On the other hand, MSS was significantly worse in positive SLN patients than in negative SLN patients, while it was not in non-identified SLN patients with respect to negative SLN patients (Figure 1). In this case, Breslow ≥ 2 mm was the most important risk factor for a shorter MSS (HR = 3.2; *p* < 0.001) (Table 3).

To the best of our knowledge, there are still no published data about the prognosis of melanoma patients with NI of SLN. However, it has been observed that the prognosis of patients with thick melanoma is similarly poor in both patients with positive SLN and in patients in whom SLNB is not performed [11,12,13]. Pavri et al. showed a significant decrease in overall survival in NV patients as compared to a random cohort of melanoma patients, but not in MSS. The authors did not evaluate the prognostic role of NI, although they reported that 44% of the patients without identification of SLN after NV developed recurrent disease, hinting that failure to intraoperatively locate SLN after NV on lymphoscintigraphy may be associated with poorer outcomes [8]. This could be biologically explained by the hypothesis that metastatic disease might lead to obstructed lymph flow, resulting, initially, in NV of the SLN and, finally, in a worse prognosis [23].

The main limitations of this study include the low percentage of nodal recurrences, and a lack of information regarding BMI in 25% of eligible patients. Another limitation is not having assessed the impact of trials with adjuvant immunotherapies on patients’ survival, although these treatments were not available for most of them (collected until 30 February 2016). The strengths of the study include its prospective collection of data, the high number of enrolled patients, and the long median follow-up time.

In light of our results, we recommend follow-up patients with NI of SLN in the same way as patients with positive SLN. Even more so, since CLND is not anymore recommended in patients with positive SLN [2,20,21]. Instead of CLND, a stricter ultrasonographic follow-up of the lymphatic drainage basins is advocated. This should be performed on both sentinel and non-sentinel lymph node basins, especially if the melanoma is localized on the trunk [10]. Indeed, other authors signaled as common practice the performance of ‘watchful waiting’ of the lymph nodes with clinical exams and ultrasound for those patients with NV who did not undergo SLNB (so, with NI of SLN) [9]. Such an approach could allow an earlier identification and management of melanoma recurrences. Larger multicentric series are needed to support our data.

## 4. Materials and Methods

### 4.1. Study Design

The present study is an observational retrospective study on patients affected by primary cutaneous melanoma who underwent SLNB.

**Figure 3:**
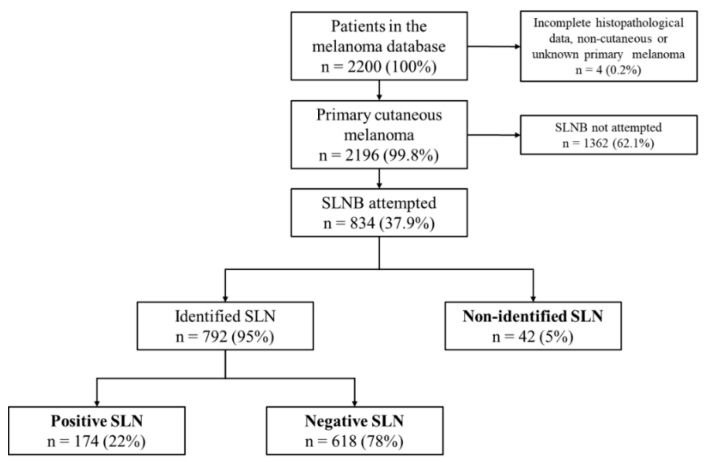
Consort diagram describing inclusions and exclusion criteria of the study. SLNB = sentinel lymph node biopsy; SLN = sentinel lymph node.

All cases of primary cutaneous melanoma undergoing SLNB from 1 January 2000 to 30 February 2016 were selected from the computerized melanoma patient database of the Dermatology Unit of the Instituto Valenciano de Oncología and included in the study. Patients with incomplete histopathological data, and non-cutaneous or unknown primary melanoma were excluded (Figure 3).

SLN status constituted the dependent variable. Thus, three main patient groups were defined: (1) positive SLN; (2) negative SLN; (3) non-identified SLN. Non-identified SLN was defined as the failure to identify SLN during SLNB, either because the surgeon chose not to attempt the procedure after NV during preoperative lymphoscintigraphy or because of a NI of the SLN intraoperatively. Therefore, no lymph nodes were collected from these patients.

Covariates selected were: age at diagnosis (<64 vs. ≥64 years), gender, obesity (BMI <30 vs. ≥30), anatomical localization (head/neck vs upper extremities vs trunk vs lower extremities vs acral), histological type (LMM vs superficial spreading vs nodular vs acral vs other/not specified), Breslow thickness (≤1.00 mm vs. 1.01–2.00 mm vs. 2.01–4.00 mm vs. >4.00 mm), ulceration status, mitotic index (≤2 mit/mm^2^ vs. >2 mit/mm^2^), presence of microscopic satellite or not, vascular invasion status, and regression status.

To evaluate the prognostic role of non-identification of SLN, we considered two outcomes: (1) the development of disease recurrence and (2) the occurrence of melanoma-specific death.

DFS was defined as the time interval between the excision of the primary tumor and the appearance of a histologically proven melanoma recurrence, both locoregional, cutaneous or nodal, than distant. MSS was defined as the time interval from the excision of the primary tumor to death from melanoma.

All patients with positive SLNB or nodal recurrence during follow-up underwent CLND unless contraindicated or refused by the patient. Follow-up was conducted depending on the melanoma stage (Table S1). However, patients with non-identified SLN underwent follow-up with ultrasonography of the regional lymphatic drainage basins every four months for two years, then every six months for five years, then annually, independently of stage.

### 4.2. Lymphoscintigraphy Procedure

Approximately 1 mCi of 99mTc-nanocolloidal albumin were injected around the scar of the previous excisional (or, rarely, incisional) biopsy with three to four intradermal injections using a 30-G needle. If the injection area was close to the expected nodal drainage site, the dose of radiotracer was halved. Then, a hand-held gamma camera was placed over the injection area and sequential images of 15 s were collected for SLN localization. Generally, the waiting time for node visualization was 1 h. In-transit nodes and nodes detected in additional drainage basins were also considered SLNs. Furthermore, if multiple nodes were visualized in the same nodal bed, the first and/or the brightest node was considered the SLN. In case of NV of the SLN, the surgeon performed a meticulous intraoperative evaluation of the expected drainage basin(s) with the gamma probe, before deciding to attempt or not the SLNB. All the lymphoscintigraphies were performed about three hours before the SLNB.

### 4.3. SLNB Procedure

Intradermal injection of blue dye around the surgical scar of melanoma was performed intraoperatively in every patient undergoing SLNB. All radiolabeled lymph nodes and/or those that appeared blue-stained during surgery were considered SLNs and were excised. After removing SLNs, the surgeon ensured that no radiolabeled or blue-stained tissue remained in the basin. SLNs were then classified depending on size. SLNs of ≤5 mm were bisected, while SLNs of >5 mm were sectioned every 2–3 mm parallel to the short axis. After a 24 h fixation in buffered formalin, SLN specimens were embedded in paraffin blocks. Finally, three histological sections were realized every 250 µm until the whole block was gone. One section was stained with hematoxylin and eosin, one with S-100, and one with Human Melanoma Black (HMB45).

### 4.4. Statistical Analysis

All the analyzed variables were expressed categorically. Differences in the distribution of each variable between the defined groups were assessed by contingency tables and the significance was analyzed by chi-squared and Fisher’s exact tests. Classification and Regression Tree (CART) analysis led to the recodification of two covariates. Anatomical localization was divided into three categories: head and neck, upper extremities, and other localization. Histological type was dichotomized into LMM and non-LMM subtypes. Univariate logistic regressions were applied to evaluate which covariates could be predictors of NI of SLN. After which, a stepwise forward multivariate logistic regression was performed using only the covariates that resulted significant (*p* < 0.05) at the univariate analysis. Survival estimates were derived by the Kaplan-Meier method, in which the event was the development of disease recurrence, locoregional nodal recurrence or the occurrence of melanoma-specific death. Patients who did not develop disease recurrence, nodal recurrence or did not die of melanoma at the last date of follow-up or date of death by other causes were censored. Differences in survival in each group (negative SLN, positive SLN, non-identified SLN) were tested by the log-rank test. The size of the effect on survival due to each variable was first explored by univariate Cox proportional hazards method. Then, stepwise forward multivariate Cox proportional hazards regressions were carried out. Only the variables with a *p* < 0.05 were entered in the multivariate models. For these models, the multiple imputation function was used to replace missing data values. This assumed that missing values were missing at random. We created 10 complete datasets using a fully conditional specification model by means of chained equations. To generate the missing values, we used all the variables to be subsequently analyzed. To assess the convergence and stationarity of each chain, we examined imputed values against iteration numbers. The results of the complete dataset analyses were combined into a single set of estimates using Rubin rules [24]. Furthermore, Breslow thickness was recoded in two categories: <2 mm and ≥2 mm. All tests were two-sided and the level of significance was set at alpha < 0.05. Statistical analyses were performed using IBM SPSS 20.0 (IBM SPSS Statistics, Chicago, IL, USA).

### 4.5. Ethics

According to our national regulations, the confidentiality of all patient information was maintained. All patients gave written permission to be included in our database and to participate in our study (Comité de Ética de la Investigación de la Fundación Instituto Valenciano de Oncología (CEI-FIVO): 2014-51).

## 5. Conclusions

The phenomenon of NI of SLN has not been thoroughly evaluated (so far). 5% of our SLNB resulted in NI of SLN. The age at diagnosis (≥64 years), obesity (BMI ≥ 30), and head and neck localization were found to be predisposing factors. Non-identified SLN patients had worse nodal DFS compared to negative SLN patients, but not worse MSS. Remarkably, they showed an HR for nodal recurrences higher than positive SLN patients. Our findings suggest to follow-up patients with non-identified SLN in the same way as patients with positive SLN.

## Figures and Tables

**Table 1:** Patients’ characteristics and their correlation with sentinel lymph node (SLN) status.

	Overall Population		Identified SLN						Non-Identified SLN			
			Total		+SLN		−SLN					
	n	%	n	%	n	%	n	%	n	%	*p* *	*p* **
**Age at diagnosis (m.v. = 0)**											<0.001	<0.001
<64 years old	549	65.8	532	67.2	108	62.1	424	68.6	17	40.5		
≥64 years old	285	34.2	260	32.8	66	37.9	194	31.4	25	59.5		
**Gender (m.v. = 0)**											0.75	0.54
Male	439	52.6	418	52.8	98	56.3	320	51.8	21	50		
Female	395	47.2	418	47.2	76	43.7	298	48.2	21	50		
**BMI (m.v. = 240)**											0.007	0.025
<30	476	80.1	457	81.2	89	80.2	368	81.4	19	61.3		
≥30	118	19.9	106	18.8	22	19.8	84	18.6	12	38.7		
**Anatomical loc. (m.v. = 0)**											0.80	<0.001
Head/neck	127	15.2	103	13	15	8.6	88	14.2	24	57.1		
Upper extremities	121	14.5	114	14.4	24	13.8	90	14.6	7	16.7		
Trunk	337	40.4	330	41.7	79	45.4	251	40.6	7	16.7		
Lower extremities	163	19.5	160	20.2	29	16.7	131	21.2	3	7.1		
Acral	86	10.3	85	10.7	27	15.5	58	9.4	1	2.4		
**Histological type (m.v. = 0)**											<0.001	<0.001
Lentigo maligna melanoma	32	3.8	24	3	0	0	24	3.9	8	19		
Superficial spreading	474	56.8	452	57.1	80	46	372	60.2	22	52.4		
Nodular	221	26.5	211	26.6	72	41.4	139	22.5	10	23.8		
Acral	45	5.4	44	5.6	13	7.5	31	5	1	2.4		
Other/not specified	62	7.4	61	7.7	9	5.2	52	8.4	1	2.4		
**Breslow thickness (m.v. = 0)**											0.61	<0.001
≤1.00 mm	225	27.0	217	27.4	13	7.5	204	33	8	19.0		
1.01–2.00 mm	297	35.6	282	35.6	45	25.9	237	38.3	15	35.7		
2.01–4.00 mm	189	22.7	178	22.5	68	39.1	110	17.8	11	26.2		
>4.00 mm	123	14.7	115	14.5	48	19	67	10.8	8	19.0		
**Ulceration (m.v. = 23)**											0.92	<0.001
Present	226	27.9	214	27.8	94	55.3	461	77	12	28.6		
Absent	585	72.1	555	72.2	76	44.7	138	23	30	71.4		
**Mitotic index (m.v. = 211)**											0.45	<0.001
≤2 mit/mm^2^	350	56.2	331	56.5	43	32.8	288	63.3	19	51.4		
>2 mit/mm^2^	273	43.8	255	43.5	88	67.2	167	36.7	19	48.6		
**Microscopic satellites (m.v. = 66)**											0.36	0.52
Absent	745	97	705	97.1	153	96.8	552	97.2	40	95.2		
Present	23	3	21	2.9	5	3.2	16	2.8	2	4.8		
**Vascular invasion (m.v. = 66)**											0.64	0.01
Absent	743	96.7	703	96.8	146	93	557	97.9	40	95.2		
Present	25	3.3	23	3.2	11	7	12	2.1	2	4.8		
**Regression (m.v. = 66)**											0.37	0.02
Absent	641	86.6	604	86.4	139	93.3	465	84.5	37	90.2		
Present	99	13.4	95	13.6	10	6.7	85	15.5	4	9.8		

m.v. = missing values; BMI = body mass index; loc. = localization. * *p* value from chi-squared of Pearson (or Fisher where appropriate) test comparing patients with identified SLN and patient with non-identified SLN. ** *p* value from chi-squared of Pearson test comparing positive SLN, negative SLN and non-identified SLN patients.

**Table 2:** Univariate and stepwise forward multivariate logistic regression models analyzing variables associated to non-identification of the sentinel lymph node.

	Univariate			Multivariate with BMI *			Multivariate without BMI		
	OR	CI 95%	*p*	OR	CI 95%	*p*	OR	CI 95%	*p*
**Age at diagnosis**									
<64 years	Ref.	Ref.	-	Ref.	Ref.	-	Ref.	Ref.	-
≥64 years	3.0	1.6–5.7	0.001	2.9	1.3–6.6	0.009	2.2	1.1–4.2	0.021
**BMI**									
<30	Ref.	Ref.	-	Ref.	Ref.	-	-	-	-
≥30	2.7	1.3–5.8	0.009	3.8	1.6–9.0	0.002	-	-	-
Anatomical localization									
Head/neck	12.2	5.8–25.6	<0.001	17.5	7.1–43.1	<0.001	12.2	5.8–25.6	<0.001
Upper extremities	3.2	1.2–8.5	0.018	2.8	0.8–9.7	0.111	3.2	1.2–8.5	0.018
Other location	Ref.	Ref.	-	Ref.	Ref.	-	Ref.	Ref.	-
**Histological type**									
LMM	7.5	3.2–18.0	<0.001	-	-	-	-	-	-
Non-LMM	Ref.		-	-	-	-	-	-	-

OR = odds ratio; CI = confidence interval; BMI = Body Mass Index; Ref. = reference category; LMM = lentigo maligna melanoma. * In this model 240 (29%) cases were excluded due to missing information for BMI.

**Table 3:** Stepwise forward multivariate Cox proportional hazards regressions of variables associated to worse nodal disease-free survival (DFS) and melanoma-specific survival (MSS).

	Nodal DFS			MSS		
	HR	CI 95%	*p*	HR	CI 95%	*p*
**SLN**						
Negative	Ref.	Ref.	-	Ref.	Ref.	-
Positive	3.2	1.4–7.5	0.006	2.9	1.9–4.3	<0.001
Non-identified	5.1	1.6–16.2	0.006	1.2	0.5–3.1	0.665
**Breslow**						
<2 mm	Ref.	Ref.	-	Ref.	Ref.	-
≥2 mm	4.2	1.7–10.4	0.002	3.2	2.0–4.7	<0.001
**Vascular invasion**						
Absent	Ref.	Ref.	-	-	-	-
Present	3.7	1.2–11.2	0.022	-	-	-
**Age at diagnosis**						
<64 years	-	-	-	Ref.	Ref.	-
≥64 years	-	-	-	2.3	1.6–3.3	<0.001
**Microscopic satellites**						
Absent	-	-	-	Ref.	Ref.	-
Present	-	-	-	2.7	1.3–5.7	0.009

HR = hazard ratio; CI = confidence interval; SLN = sentinel-lymph node; Ref. = reference category.

## Supplementary Materials

The following are available online at https://www.mdpi.com/2072-6694/12/11/3151/s1, Table S1: Follow-up of patients per disease stage.
