# Supplementary material for: Prognostic Role of Non-Identification of Sentinel Lymph Node in Cutaneous Melanoma Patients: An Observational Retrospective Study
Source: Cancers (Basel). 2020 Oct 27;12(11):3151. doi: 10.3390/cancers12113151 (PMC7692392; doi:10.3390/cancers12113151)
Supplement: Supplementary file 1 [file cancers-12-03151-s001.pdf]

cancers

Supplementary Material

Prognostic Role of Non-Identification of Sentinel Lymph Node in Cutaneous Melanoma Patients: An Observational Retrospective Study

Ruggero Moro, Cintia Arjona-Aguilera, Celia Requena, Virginia Pont-Sanjuan, Victor Traves, Esperanza Manrique-Silva and Eduardo Nagore

Table S1. Follow-up of patients per disease stage.

Stage	TBSE + H&P	NBU	CT total body	MR of CNS
0	12 months interval	-	-	-
IA	6 months interval for 5 years, then annually	-	-	-
IB	6 months interval for 5 years, then annually	Annually, or depending on physical exploration	-	-
IIA-B	6 months interval for 5 years, then annually	6 months interval for 3 years, then annually, or depending on physical exploration	-	-
IIC/IIIA	6 months interval for 5 years, then annually	6 months interval for 5 years, or depending on physical exploration	6 months interval for 5 years	-
IIIB-C	3 months interval for 2 years, then 6 months interval for 3 years, then annually	6 months interval for 5 years or depending on physical exploration	6 months interval for 5 years	Annually for 5 years
IV NED	3 months interval for 3 years, then 6 months interval for 2 years, then annually	Depending on physical exploration	3 months interval for 1 year, then 6 months interval for 4 years, then annually	Annually and before surgery of CNS metastases

TBSE = total body skin examination with dermoscopic evaluation of cutaneous lesions; H&P = history and physical exam with palpation of nodal basins; NBU = nodal basins ultrasound; TC = computed tomography; MR = magnetic resonance; CNS = central nervous system
